# A transcription factor TaMYB5 modulates leaf rolling in wheat

**DOI:** 10.3389/fpls.2022.897623

**Published:** 2022-08-23

**Authors:** Zhi Zhu, Jingyi Wang, Chaonan Li, Long Li, Xinguo Mao, Ge Hu, Jinping Wang, Jianzhong Chang, Ruilian Jing

**Affiliations:** ^1^Shanxi Institute of Organic Dryland Farming, Organic Dry Farming of Shanxi Province Key Laboratory, Shanxi Agricultural University, Jinzhong, China; ^2^National Key Facility for Crop Gene Resources and Genetic Improvement, Institute of Crop Sciences, Chinese Academy of Agricultural Sciences, Beijing, China; ^3^College of Agronomy, Shanxi Agricultural University, Jinzhong, China

**Keywords:** GWAS, leaf rolling, TaMYB5, *NRL1*, canopy temperature, wheat

## Abstract

Leaf rolling is an important agronomic trait in wheat (*Triticum aestivum* L.). Moderate leaf rolling keeps leaves upright and maintains the relatively normal photosynthesis of plants under drought stress. However, the molecular mechanism of wheat leaf rolling remains unclear. Here, we identified a candidate gene *TaMYB5-3A* that regulates leaf rolling by using a genome-wide association study (GWAS) in a panel of 323 wheat accessions. Phenotype analysis indicated that the leaves of *tamyb5* mutants were flatter than that of the wild type under drought condition. A nucleotide variation in the *TaMYB5-3A* coding region resulted in a substitution of Thr to Lys, which corresponds to two alleles *SNP*-3A-1 and *SNP*-3A-2. The leaf rolling index (LRI) of the *SNP*-3A-1 genotype was significantly lower than that of the *SNP*-3A-2 genotype. In addition, *TaMYB5-3A* alleles were associated with canopy temperature (CT) in multiple environments. The CT of the *SNP*-3A-1 genotype was lower than that of the *SNP*-3A-2 genotype. Gene expression analysis showed that *TaMYB5-3A* was mainly expressed in leaves and down-regulated by PEG and ABA treatment. TaMYB5 induces *TaNRL1* gene expression through the direct binding to the AC *cis*-acting element of the promoter of the target gene, which was validated by EMSA (electrophoretic mobility shift assay). Our results revealed a crucial molecular mechanism in wheat leaf rolling and provided the theoretical basis and a gene resource for crop breeding.

## Introduction

Wheat (*Triticum aestivum*L.) has been one of the most widely cultivated crops in the world since the Neolithic Age and provides about 20% of calorie intake for humans (Shiferaw et al., 2011). Leaf is the primary organ to process photosynthesis in plants, and the photosynthetic rate is directly affected by its morphological characteristics (Zhang et al., 2015). Leaf shape plays a critical role in photosynthesis and plant development by affecting light interception, transpiration, and leaf dehydration (O”Toole and Cruz, 1980). Leaf rolling is a highly complex agronomic trait regulated by genotype and environment (Li et al., 2017). Moderate leaf rolling keeps the leaves upright and improves the population structure, finally enhances light capture and photosynthesis (Zhu et al., 2001; Richards et al., 2002). Under drought stress, moderate leaf rolling maximizes light capture, accelerates dry-matter accumulation, boosts yield, decreases transpiration, changes water uptake capacity, and then affects drought resistance (Lang et al., 2004; Moon and Hake, 2011). In addition, appropriate leaf rolling can decrease the solar radiation on leaves, cumulative transpiration, and water loss, and thus leaf rolling is crucial to improve tolerance of plants to drought stress (Lian et al., 2004; Kadioglu and Terzi, 2007). Therefore, leaf rolling is regarded as a crucial agronomic trait for crop breeding, which is extremely significant for obtaining ideal plant architecture and drought tolerance (Yuan, 1997; Chen et al., 2001).

Due to its agronomic importance, some genes and their pathways involved in controlling leaf rolling have been identified and characterized in crop plants. Most genes could regulate leaf rolling by affecting bulliform cells development or secondary cell wall biosynthesis, for example, *srl1* and *url1* mutants exhibited adaxial rolling of leaves due to changes in the number and size of bulliform cells (Xiang et al., 2012; Fang et al., 2021). *psl1* is a rice photo-sensitive leaf rolling mutant that bulliform cells and cell wall biosynthesis changed significantly, which displayed leaf rolling when exposed to high light intensity (Zhang et al., 2021). *NRL1* encodes a cellulose synthase-like protein that is crucial for cell wall formation, and the reduced expression of *NRL1* in the mutant displayed adaxially rolled leaves, which was due to the reduction of bulliform cells size in rice (Hu et al., 2010). The loss-of-function mutant of *rl14* and *cld1* displayed leaf rolling *via* affecting bulliform cells size, and the cell wall formation was affected by changing the content of lignin and cellulose in the cell wall. The loss of *CLD1* function accelerated water loss, reduced leaf water content, and drought resistance of the plant (Fang et al., 2012; Li et al., 2017). The genes of *ACL1* and *ROC5* have also been reported to regulate leaf rolling by affecting bulliform cell development (Li et al., 2010; Zou et al., 2011). Moreover, the abnormal establishment of adaxial–abaxial polarity also is considered a crucial factor in leaf rolling (Yamaguchi et al., 2012). A maize gene *RLD1* encodes an HDZIP III family protein that regulates leaf abaxial establishment (Juarez et al., 2004). Recent studies have shown that *rolled fine striped* (*RFS*) gene causes extreme leaf rolling due to the abnormal development of vascular cells on the adaxial side of leaf in rice (Cho et al., 2018). *SLL1* and *SRL2* were also found to regulate leaf rolling by influencing the development of abaxial sclerenchymatous cells in rice (Liu et al., 2016).

MYB transcription factors (TFs) contain a highly conserved MYB DNA-binding domain (DBD) that generally consists of 1–4 incomplete MYB repeats of approximately 52 amino acids. Based on the number of incomplete repeats in their DBD, the MYB family is divided into four classes, 1R-MYB, R2R3-MYB, 3R-MYB, and 4R-MYB proteins. MYB TFs play an essential role in regulating various developmental processes, primary and secondary metabolism, and a series of stress responses in plants (Dubos et al., 2010; Li et al., 2019a). Interestingly, the MYB TFs are also involved in regulating leaf rolling, such as *SLL1* encodes a SHAQKYF class MYB protein belonging to the KAN subfamily and regulates leaf rolling by promoting the development of sclerenchymatous cells. The loss-of-function mutant *sll1* displayed defective sclerenchymatous cells due to programmed cell death of the mesophyll cells on the abaxial side (Zhang et al., 2009). In addition, one of the most likely candidate genes in the rolled-leaf mutant (*rl10*) was also the MYB transcription factor belonging to the KANADI subfamily (Luo et al., 2007). The genes of *TaMYB18*, *OsMYB60*, and *AtMYB101* have also been reported to regulate leaf rolling (An et al., 2014; Zhang et al., 2016; Liu et al., 2018). Moreover, the MYB TFs regulated leaf rolling by affecting secondary cell wall biosynthesis. Overexpression of *OsMYB103L* showed that the cellulose content and the expression levels of several cellulose synthase genes (CESAs) were remarkably increased, and resulted in the rolling of leaves in rice (Yang et al., 2014). Recent studies have shown that *OsSND2* regulates leaf rolling by promoting the expression of *OsMYB61*, which affects the biosynthesis of secondary cell walls (Ye et al., 2018).

Several genes that regulate leaf rolling in crops have been reported, but their molecular mechanisms, especially in wheat, remain unclear. In the present study, through genome-wide association study (GWAS), we identified a novel R2R3-MYB gene, *TaMYB5*, that regulates leaf rolling in wheat. Association analysis showed that *TaMYB5* was significantly associated with canopy temperature (CT) in multiple environments, and favorable genotypes of *TaMYB5* have been positively selected in different ecological regions of wheat in China. Further studies showed that TaMYB5 directly binds to the promoter through AC *cis*-acting element to induce the expression of the *TaNRL1* gene. The phenotype of *tamyb5* mutants under drought stress verified that *TaMYB5* was involved in leaf rolling regulation. Our results revealed a crucial molecular mechanism of wheat leaf rolling, and provided the theoretical basis and a gene resource for crop breeding.

## Materials and methods

### Plant materials and measurement of leaf rolling index

Thirty-two wheat accessions with wide variations (Zhang et al., 2013) were used to detect the nucleotide polymorphism of the target gene. Three winter wheat populations were employed in this study. Population 323 (P323) was used for GWAS. The accessions include 12 landraces, 36 advanced lines, and 275 modern cultivars, most of whose flowering dates occurred within 1 week, mainly planted in the Yellow and Huai River Valleys Facultative Wheat Zone and the Northern Winter Wheat Zone in China (Li et al., 2019b). Population 157 (P157) (157 landraces) and Population 348 (P348) (348 modern cultivars) collected from all 10 wheat production zones in China were used for haplotype analysis (Wang et al., 2019).

The P323 was planted to investigate agronomic traits at Shunyi (SY) (40°23′N; 116°56′E) and Changping (CP) (40°13′N; 116°13′E) in Beijing, China during the 2016–2017 growing seasons. Two water treatments, rain-fed (drought stress, DS) and well-watered (WW), were applied. The WW plot was irrigated with 750 m^3^ ha^–1^ (75 mm) water at pre-overwintering, flowering, and grain-filling stages, respectively, if the rainfall in the corresponding period was insufficient. The total precipitation in the growing seasons was 143 mm. Heat stress (HS) treatment was conducted in a polyethylene-covered greenhouse at the 1-week post-anthesis stage (Li et al., 2019b). Each accession was planted in four rows, with row spacing of 30 cm, length of 2 m, and 40 seeds in each row. Five plants were randomly selected for phenotyping.

For GWAS, P323 was planted in Changping, Beijing, during the 2019–2020 and 2020–2021 growing seasons, and the LRI of 323 accessions was measured. Two water regimes DS and WW were applied in this study. The width of the flag leaf was detected one-third away from the leaf sheath, and the natural leaf width (Ln) and fully unfolded leaf width (Lw) were measured with three replicates, respectively. The LRI data were measured from 8:00 to 10:00 and from 15:00 to 18:00 at the 2-week after flowering. The LRI of flag leaves was calculated by the formula: “LRI = [(Lw − Ln)/Lw] × 100%,” Lw means the expanded distance of leaf blade margins, Ln means the natural distance of the leaf blade margins, and the low and high of LRI value represent the slight and severe degree of leaf rolling, respectively (Shi et al., 2007).

### Genome-wide association study of leaf rolling

GWAS of wheat leaf rolling genes was performed by analyzing the wheat natural variation population P323. The overall LRI used for GWAS was calculated as the best linear unbiased predictions (BLUPs), which were based on a mixed linear model and performed using the lme4 package in R 3.3.0. Genotyping was performed by using the 660K SNP Array as previously described (Li et al., 2019b). After removing nucleotide variations with missing rates ≥ 0.2 and minor allele frequency (< 0.05), 395,675 SNPs were used for GWAS in this research. On the whole genome scale, in consideration of the population structure (Q) and familial kinship (K), a mixed linear model (MLM) was applied to analyze the association between molecular markers and traits by using TASSEL 5.0 software (Li et al., 2019c).

### Generation and phenotypic analysis of wheat mutant

Three wheat mutants (*tamyb5*) were obtained by exome sequencing (data not published) from a population, which was a wheat variety Aikang58 (AK58) mutant population induced by ethyl methanesulfonate (EMS). These mutants were backcrossed two times with wild-type AK58 to eliminate the noise in the genetic background. To observe the leaf phenotype at the flowering stage, wheat plants were grown in artificial climate chambers under long-day conditions (16 h light, 25°C/8 h dark, 20°C) at 60% humidity. Plants were treated with drought stress at the post-anthesis stage. The leaf phenotyping was performed and the LRI was measured after 18 days of drought treatment.

### Functional marker development and association analysis

Wheat DNA was extracted from the young leaves by the CTAB method (Stewart and Via, 1993). *TaMYB5* was cloned by performing PCR amplification using TransStart FastPfu DNA Polymerase and subsequently ligated into a *pEASY*-Blunt vector for sequencing (*Trans* Gen Biotech, Beijing). The PCR conditions were 95°C for 5 min; 35 cycles of 95°C for 30 s, 60°C for 30 s, 72°C for 1 min; followed by a final extension of 72°C for 10 min. Five positive clones were randomly picked for sequencing.

Sequence polymorphism of the *TaMYB5* gene was detected in 32 wheat accessions with wide variations. Based on a SNP (C/A) located at 544 bp of the gene coding region, a dCAPS marker was developed to distinguish the C/A genotype by mismatching nucleotide to create the *Sal* I restriction site. The first-round PCR products were obtained by genome-specific primers and used as the templates for the second round of PCR. Second-round PCR products were digested by *Sal* I restriction enzyme at 37°C for 2 h and then target fragments were separated by electrophoresis in 4% agarose gel (Wang et al., 2020). Association analyses were conducted by SPSS 16.0 software. *P* < 0.05 was considered a significant correlation. The primers are shown in Supplementary Table 1.

### Gene expression analysis

Gene expression analysis was performed using wheat variety Hanxuan 10 as the plant material. Tissue-specific gene expression was analyzed in leaf, root base, and root of seedling at the two-leaf stage, as well as in leaf, spike, penultimate node, root base, root tissues at different depths of jointing stage plants. Wheat seedlings were cultured by hydroponics. Seedlings at two-leaf stage were treated with PEG-6000 (−0.5 MPa) and 50 μM abscisic acid (ABA), and the leaves were collected at 0, 0.5, 1, 2, 3, 6, 12, 24, 48, and 72 h under the treatments (20 h light/4 h dark). Samples at 0 h served as the control. Total RNA was extracted with a TRIzol reagent (TIANMO, Beijing). cDNA was synthesized with a FastQuant RT Kit (TIANGEN, Beijing). Gene expression levels in plant samples were detected using quantitative real-time PCR (qRT-PCR), which was performed with a SYBR Green PCR Master Mix Kit (TAKARA, Beijing). Five plants were harvested for each replicate, and the three biological replicates were set for each treatment.

### Subcellular localization

Full-length ORF of *TaMYB5* was cloned and inserted into the pCAMBIA1300GFP vector to generate the p35S-TaMYB5-1300GFP construct. The recombinant plasmid was transformed into *Agrobacterium tumefaciens* strain EHA105 and then transiently expressed into *Nicotiana benthamiana* leaves (Zhuang et al., 2021). After 48 h of culture, the green fluorescence signal was detected by a confocal microscope (Carl Zeiss, Germany).

### Transcriptional activity analysis

To detect the transcription activity, the full-length cDNA of *TaMYB5* was fused into p35S-GAL4DB-NOS to generate the p35S-GAL4-TaMYB5-NOS fusion plasmid. The p35S-5 × GAL4-LUC-NOS, p35S-REN-NOS vectors served as the reporter plasmids, and p35S-GAL4DB-NOS served as the effector plasmids (Liu et al., 2020). The TaMYB5-GAL4DB, the firefly luciferase (LUC), and Renilla luciferase (REN) plasmids were co-transformed into tobacco protoplasts to detect the LUC activity ratios (LUC/REN).

### Dual-luciferase reporter assay

The p35S-TaMYB5-1-1300GFP and p35S-TaMYB5-2-1300GFP were used as effectors. *proTaNRL1* was fused into the pGreenII 0800-LUC vector to generate the reporter constructs. pCAMBIA1300-GFP served as vector control. They were transformed into *Agrobacterium tumefaciens* strain GV3101 and then transiently expressed into tobacco leaves (Li et al., 2022). The luciferase activities were tested using Dual-Glo Luciferase Assay System (Promega, United States).

### Yeast one-hybrid assays

The CDS sequences of *TaMYB5-1* and *TaMYB5-2* were cloned and fused into the pB42AD as the effector vector. The promoter fragment of *TaNRL1* containing the “ACCTAAC” element was cloned and inserted into the pLacZ vector as a reporter. The effector and reporter plasmids were co-transformed into yeast strain EGY48. Empty pB42AD and pLacZ were used as negative controls. Yeast strain was grown on SD/-Trp/-Ura medium containing X-Gal to detect the interaction at 29°C.

### Electrophoretic mobility shift assays

The cDNA sequence of *TaMYB5* was fused into vector pGEX-4T1 to construct GST-TaMYB5. The empty GST vector pGEX-4T1 and GST-TaMYB5 were transformed into the *Escherichia coli* expression strain BL21 (Wang et al., 2020). TaMYB5 protein was induced by 0.2 mM IPTG at 16°C for 10 h and was purified using glutathione-sepharose 4B (GE Healthcare, United States). Biotin-labeled ACC-box and mutant ACC-box, unlabeled ACC-box and mutant ACC-box, and their reverse complementary sequences were synthesized and used as probes. EMSA assay was performed using a LightShift^®^ Chemiluminescent EMSA Kit (Thermo, United States).

## Results

### *TaMYB5-3A* is associated with wheat leaf rolling

**FIGURE 1:**
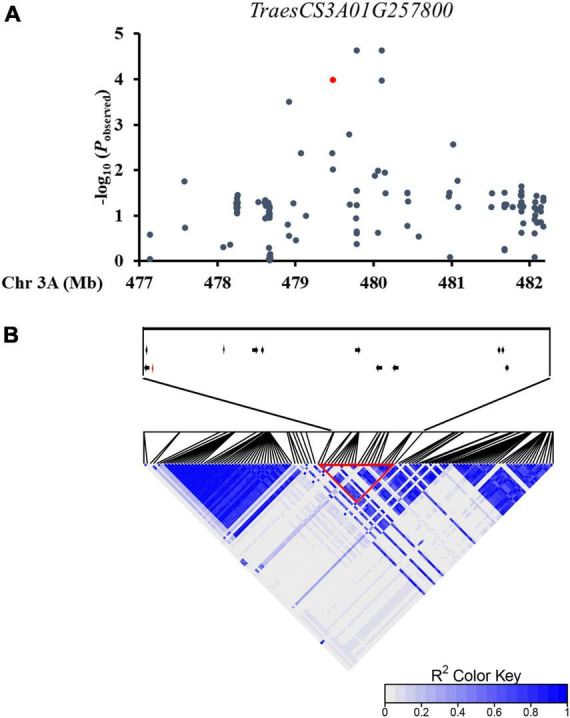
A natural variation in *TaMYB5* was associated with wheat leaf rolling. **(A)** A regional Manhattan diagram of the *TaMYB5-3A* genomic region (477,138,673 bp–482,177,523 bp). One hundred twenty-five SNPs were used in the association analysis. The SNP in the *TaMYB5* coding region is represented by a red dot. The *P*-value is shown on a −log10 scale. **(B)** Linkage disequilibrium (LD) heat map of the chromosome region. These genes within the block region are shown with arrows. The position of *TaMYB5-3A* (*TraesCS3A01G257800*) is shown with a red arrow. Red triangle box highlight the strong LD of a haplotype block.

In order to characterize genes associated with wheat leaf rolling, we assembled a natural population of P323 consisting of 323 winter wheat accessions from 10 major wheat regions in China, which had been used for GWAS (Li et al., 2019c; Wang et al., 2021). The leaf rolling degree of each accession was analyzed by measuring and calculating the LRI at the flowering stage. There was a large variation of LRI in 323 accessions, ranging from 0.17% to 70.63%, which indicates that the leaf rolling at the flowering stage is suitable for genetic analysis. Two extreme phenotypes of leaf rolling in the population are shown in Supplementary Figure 1. On the whole genome scale, in consideration of the population structure (Q) and familial kinship (K), mixed linear model (MLM) with the correction function was applied to analyze the association between markers and traits. A haplotype block (479,471,884 bp–480,575,035 bp) on chromosome 3A was significantly associated with LRI and contained 12 candidate genes (Figure 1 and Supplementary Table 2). It has been reported that the MYB family genes were involved in regulating leaf rolling in plant; therefore, *TraesCS3A02G257800* was selected as a candidate gene for regulating leaf rolling. Protein structure analysis showed that TraesCS3A02G257800 is an MYB protein with two incomplete MYB repeats, belonging to the R2R3-MYB family (Supplementary Figure 2A). Further phylogenetic analysis showed that MYB5 was the closest homologous to TraesCS3A02G257800, hence the gene was named *TaMYB5-3A* (Supplementary Figure 2B).

### Phenotype of *tamyb5* mutants

**FIGURE 2:**
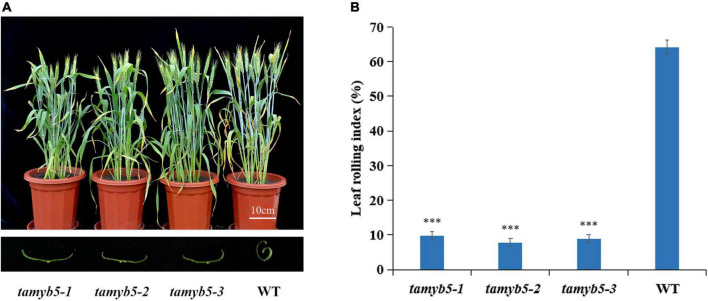
The phenotypes of *tamyb5* mutants and WT (wild-type AK58). **(A)** Phenotypes were observed under 18 days of drought treatment at the post-anthesis stage. The leaves of *tamyb5* mutants were flatter than that of the WT. There is no significant phenotypic difference in leaf rolling between *tamyb5* mutants and WT before drought stress. **(B)** Leaf rolling index (LRI) of WT and *tamyb5*. Data are presented as mean ± SE (*n* = 9). ***P < 0.001.

To verify the relationship between *TaMYB5* and wheat leaf rolling, we selected three *tamyb5* mutants (Supplementary Table 3) and backcrossed the mutants with the wild type (WT, AK58) two times to eliminate background noise. Under drought treatment, the leaves of *tamyb5* mutants were flatter than that of WT at the flowering stage (Figure 2A), and the LRI index of WT was more than 60%, while that of mutant plants was less than 10% (Figure 2B). The phenotype of the *tamyb5* mutant further confirmed that *TaMYB5* was involved in the regulation of wheat leaf rolling.

### *TaMYB5-3A* is associated with leaf rolling and canopy temperature

**FIGURE 3:**
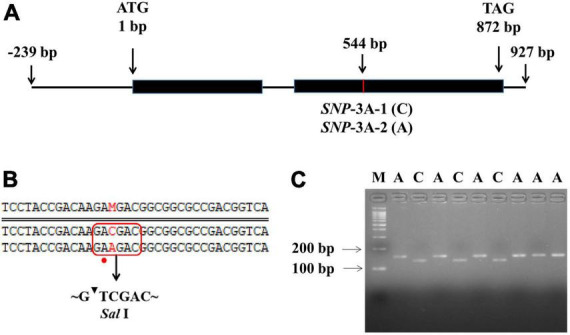
Nucleotide polymorphisms and molecular marker development of *TaMYB5-3A*. **(A)** Schematic diagram of the *TaMYB5-3A* structure, including two exons and one intron. One SNP was detected in the coding region of *TaMYB5-3A*. **(B)** The *TaMYB5-3A*-dCAPS marker was developed based on the SNP (C/A) at 544 bp. Red rectangle and dot represent the introduction of the *Sal* I restriction site by base mismatched (A to T), and red letters represent the different bases at 544 bp of the *TaMYB5-3A* coding region. **(C)** PCR products were digested by *Sal* I. The 153 bp fragment amplified from accessions with *SNP*-3A-1 could be digested by *Sal* I into 132 bp and 21 bp, while the 153 bp fragment amplified from accessions with *SNP*-3A-2 could not be digested. M, 100 bp DNA ladder. *SNP*-3A-1 **(C)** was the majority in the wheat population, and this sequence was used in subsequent experiments if not specified.

**FIGURE 4:**
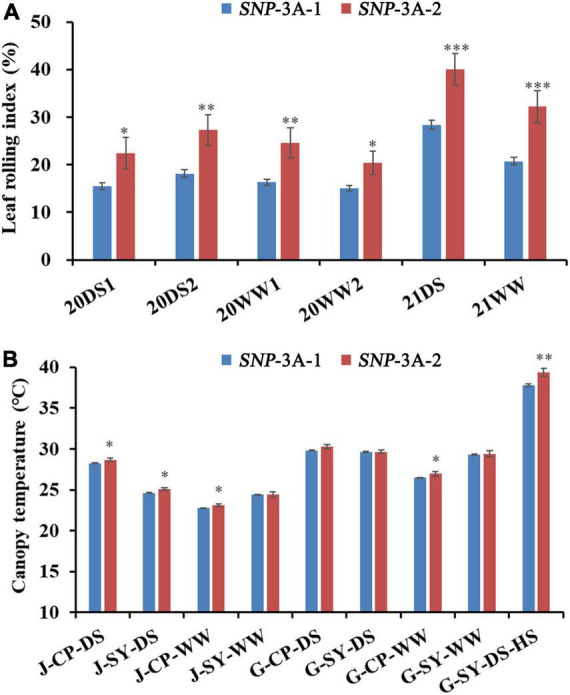
Comparisons of LRI (leaf rolling index), and CT (canopy temperature) in the two *TaMYB5-3A* genotypes *SNP*-3A-1 and *SNP*-3A-2. **(A)** LRI comparisons of two *TaMYB5-3A* genotypes in six environments. LRI data were collected in 2020 and 2021; 20, 2020; 21, 2021; DS, drought stress; WW, well-watered; 1 or 2 after 20DS or 20WW refers to two replicates set for each treatment, respectively. **(B)** CT comparisons of two *TaMYB5-3A* genotypes in nine environments. CT data were collected in 2017. Plant growth stages: J, jointing stage; G, grain-filling stage. Planting locations: CP, Changping, SY, Shunyi. Treatments: DS, drought stress; WW, well-watered; HS, heat stress. The significance of data differences is tested by Student’s *t* test. *P < 0.05; **P < 0.01; ***P < 0.001. Error bar, ± SE.

To further clarify the relationship between *TaMYB5* and leaf rolling, we analyzed the polymorphism of *TaMYB5* coding regions in 32 wheat cultivars with wide variations (Supplementary Table 4). A SNP (C/A) was detected at 544 bp of the *TaMYB5-3A* coding region, resulting in a Thr to Lys substitution, corresponding to two alleles *SNP*-3A-1 and *SNP*-3A-2 (Figure 3A). Based on the SNP (C/A), a marker of *TaMYB5-3A*-dCAPS was developed to distinguish the two alleles (Figure 3B). The marker contained a base mismatch in the upstream primer that generated the recognition site for the restriction enzyme *Sal* I (Figure 3C). *TaMYB5-3A*-dCAPS was significantly correlated with leaf rolling, and the LRI of *SNP*-3A-1 was significantly lower than that of *SNP*-3A-2 (Figure 4A). The results confirmed that *TaMYB5* played a role in wheat leaf rolling.

Previous studies suggest that leaf rolling is correlated with canopy temperature (CT) under moderate drought stress (Garrity and O’Toole, 1995). Association analysis was conducted between *TaMYB5-3A* and agronomic traits in the wheat population P323 and found that *TaMYB5-3A* was significantly correlated with CT under five environmental conditions. The CT of *SNP*-3A-1 was significantly lower than that of *SNP*-3A-2 (Figure 4B).

**FIGURE 5:**
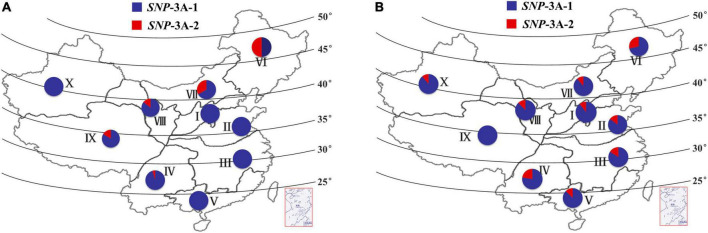
Frequency and distribution of two *TaMYB5-3A* genotypes, *SNP*-3A-1 and *SNP*-3A-2. Distribution of two *TaMYB5-3A* genotypes from 157 landraces **(A)** and 348 modern cultivars **(B)** in 10 Chinese wheat production zones. I, Northern Winter Wheat Zone; II, Yellow and Huai River Valleys Facultative Wheat Zone; III, Middle and Low Yangtze Valleys Autumn-Sown Spring Wheat Zone; IV, Southwestern Autumn-Sown Spring Wheat Zone; V, Southern Autumn-Sown Spring Wheat Zone; VI, Northeastern Spring Wheat Zone; VII, Northern Spring Wheat Zone; VIII, Northwestern Spring Wheat Zone; IX, Qinghai-Tibetan Plateau Spring-Winter Wheat Zone; X, Xinjiang Winter-Spring Wheat Zone.

The frequency distributions of these two alleles in different wheat zones were determined in wheat populations P157 (157 landraces) and P348 (348 modern cultivars). The frequency of *SNP*-3A-1 in P157 was much higher than *SNP*-3A-2 in all 10 zones except for Zone VI, especially in Zones I, II, III, V, and X where all accessions were *SNP*-3A-1 (Figure 5A). The frequency of *SNP*-3A-1 in P348 was still higher than *SNP*-3A-2 in all 10 zones (Figure 5B). However, from landraces to modern cultivars in China, *SNP*-3A-1 with flat leaf and low CT was positively selected in semi-arid areas, including the wheat Zones VI, VII, VIII, and IX, which might have improved drought tolerance of wheat accessions. *SNP*-3A-2 with rolling leaf and high CT was positively selected in humid and semi-humid areas, such as the wheat Zones I, II, III, IV, and V, which selected for moderate leaf rolling might contribute to photosynthesis in plant development (Figure 5 and Supplementary Figure 3; Zeng et al., 2011). Therefore, the favorable genotype was positively selected in areas with different rainfall levels during the process from landraces to modern cultivars in China.

### Expression pattern of *TaMYB5* gene

**FIGURE 6:**
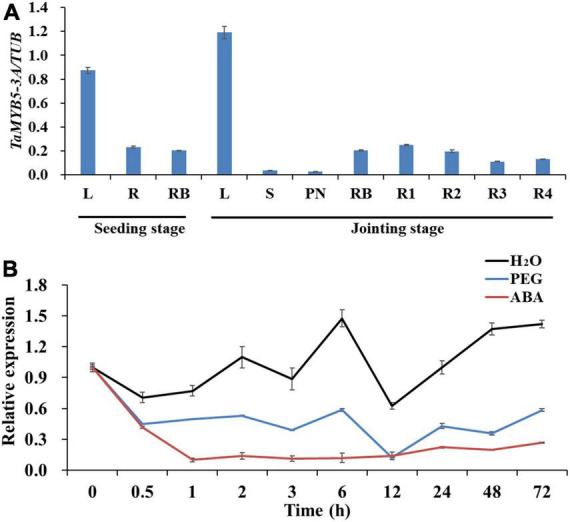
Expression patterns of *TaMYB5* in wheat. **(A)** Expression patterns of *TaMYB5-3A* in different plant tissues at seedling and jointing stage. L, leaf; S, stem; PN, penultimate node; RB, root base; R1, R2, R3, and R4 indicate the root section from the ground to 30, 30–60, 60–90, and 90–120 cm depth, respectively. **(B)** Relative expression of *TaMYB5-3A* following H_2_O (Control), PEG, and ABA treatment. Two-week-old seedlings of Hanxuan 10 were treated with PEG-6000 and 50 μM ABA. Leaves were collected for expression experiments. All data are the means ± SE of three independent experiments.

To explore the role of *TaMYB5* in plant growth and development, we detected the expression levels of *TaMYB5* in wheat tissues at the seedling stage and jointing stage by real-time quantitative PCR. The results showed that *TaMYB5* was widely expressed in all tissues, and was mainly expressed in the leaf (Figure 6A), suggesting that *TaMYB5* has a function in wheat leaf.

We next performed qRT-PCR to determine whether *TaMYB5* is affected by PEG and ABA. The results showed that both ABA and PEG treatments reduced the expression of *TaMYB5* in leaves. Under PEG treatment, the expression of *TaMYB5* was down-regulated at 0.5 h, and there was no significant change in subsequent expression (Figure 6B). The expression of *TaMYB5* decreased significantly after 0.5 h ABA treatment (Figure 6B). Taken together, these results suggest that *TaMYB5* may play an important role in plant drought tolerance.

### TaMYB5-3A is a nucleus-localized transactivator

**FIGURE 7:**
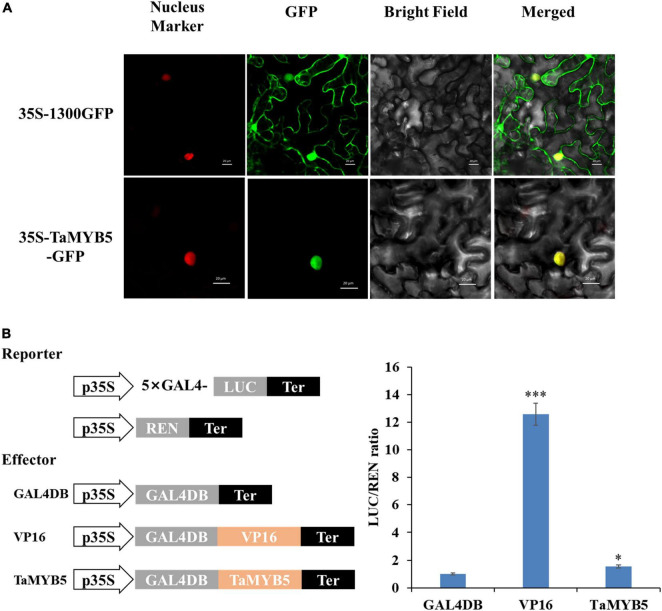
Subcellular localization and transcriptional activity analysis of TaMYB5. **(A)** 35S-TaMYB5-GFP and the nuclear localization marker (mCherry) were transiently co-transformed into tobacco leaf cells. Scale bar = 20 μm. **(B)** Transcriptional activity of TaMYB5. The LUC/REN ratio of TaMYB5. Data are means of three independent experiments. *P < 0.05, ***P < 0.001. Error bar, ± SE.

To examine the subcellular localization of TaMYB5, we constructed the fusion protein of 35S-TaMYB5-GFP and transiently expressed it in tobacco leaves. The laser scanning confocal microscope images showed that the fluorescence signal of the 35S-TaMYB5-GFP fusion protein was only detected in the nucleus; nevertheless, the fluorescence of the 35S-1300GFP vector was detected across the whole cell (Figure 7A). Moreover, TaMYB5-2 was also localized in the nucleus (Supplementary Figure 4). The result provided that *TaMYB5* encoded a nuclear localization protein, which was consistent with its potential localization as a transcription factor.

We further used a dual-luciferase reporter system to detect the transcriptional activity of TaMYB5. The empty GAL4DB vector was used as the negative control, and the fusion vector of VP16 (Herpes Simplex Virus16) was used as the positive control. As shown in Figure 7B, TaMYB5 exhibited stronger transcriptional activity than the empty GAL4DB vector. Together, these data indicate that TaMYB5 is a transcriptional activator of nuclear localization.

### *NRL1* is a putative target gene promoted by MYB5

**FIGURE 8:**
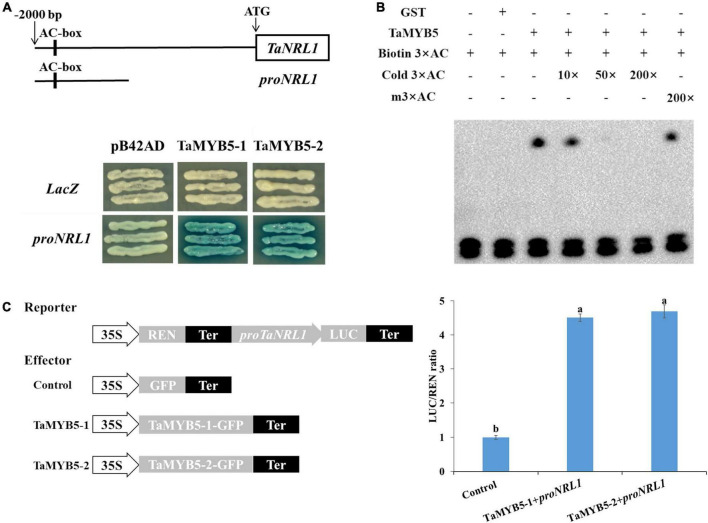
TaMYB5 binds to the wheat *TaNRL1* promoter region. **(A)** TaMYB5 activated expression in yeast of the LacZ reporter gene driven by the AC box of *TaNRL1* promoter. *proNRL1* are base pairs from 1,150 bp to 2,000 bp in the promoter of *TaNRL1*. LacZ and pB42AD were used as negative controls. TaMYB5-1 and TaMYB5-2 correspond to *SNP*-3A-1 and *SNP*-3A-2 amino acid sequences, respectively. **(B)** EMSA (electrophoretic mobility shift assay) of the TaMYB5-GST protein and the AC box probe. The lower bands show the free probes and the upper bands show that the TaMYB5-GST protein binds to the biotin-labeled AC box probe. **(C)** Dual-luciferase assay of transformed tobacco leaves to detect the interaction between TaMYB5 and the *proNRL1*. Schematic diagrams of the effector and reporter constructs are shown. Data are means (± SE) of three biological replicates.

To determine the downstream target gene of *TaMYB5*, TaMYB5-1 and TaMYB5-2 were separately constructed into the pB42AD vector, and the promoters of predicted downstream genes were cloned into the pLacZ vector for yeast one-hybrid assays. We examined whether TaMYB5 binds to the promoter of genes involved in leaf rolling regulation, namely, *ACL1*, *CLD1*, *ROC5*, *CESAs*, *RL14*, *NRL1*, *PSL1*, and *SRL2*. Yeast one-hybrid assay showed that both TaMYB5-1 and TaMYB5-2 could bind to and activate the *proNRL1* (Figure 8A). A previous study has demonstrated that *NRL1* is involved in leaf rolling regulation in rice, and the loss-of-function mutant *nrl1* displayed leaf rolling by affecting bulliform cell size (Hu et al., 2010). Yeast one-hybrid assay suggested that TaMYB5 could bind to the *proTaNRL1*, and the A/C nucleotide variation of the *TaMYB5* coding region did not affect the binding to *proNRL1*.

The AC box (ACCTAAC) is a specific *cis*-acting element regulated by the R2R3-MYB transcription factor (Prouse and Campbell, 2012). Notably, the *TaNRL1* promoter region contains the AC element (Figure 8A). After the induction and purification of the TaMYB5 protein (Supplementary Figure 5), electrophoretic mobility shift assay (EMSA) was performed to examine whether the TaMYB5 protein binds to the AC element in the *TaNRL1* promoter (Figure 8B). The migration rate of the biotin-labeled AC probe was significantly reduced with the addition of TaMYB5-GST protein, whereas the addition of GST protein alone did not affect the migration rate of the AC probe. In the competition experiment, the unlabeled AC probe could compete with the biotin-labeled AC probe, while the mutated probe did not affect the binding of the biotin-labeled probe to TaMYB5 protein, suggesting that TaMYB5 could specifically bind to the AC *cis*-acting elements.

We also performed a dual-luciferase reporter assay to further verify that TaMYB5 binds to the *TaNRL1* promoter and regulates its transcription. The p35S-TaMYB5-1-1300GFP and p35S-TaMYB5-2-1300GFP were constructed as effectors, and the *TaNRL1* promoter was inserted into the LUC vector as a reporter. Compared with the negative control, the LUC activities were higher when the effector and reporter were co-expressed in tobacco leaves (Figure 8C). However, the LUC activity between the two alleles of *TaMYB5* was not a significant difference. Taken together, the results suggest that TaMYB5 directly binds to its promoter to promote *TaNRL1* expression through AC *cis*-acting element, while the A/C variation of the *TaMYB5* coding region does not affect the binding to the *TaNRL1* promoter.

## Discussion

Leaf is the main organ of photosynthesis in wheat, and its morphology directly affects the photosynthetic rate. Moderate leaf rolling can keep the leaves upright, improve the population structure, and finally enhance the light use efficiency and yield of wheat (Richards et al., 2002; Zhang et al., 2015). Therefore, it is important to reveal the molecular mechanism of wheat leaf rolling. Here, we identified a novel gene *TaMYB5* in wheat involved in leaf rolling by using GWAS. A SNP identified in the *TaMYB5* coding region was significantly associated with leaf rolling, and the degree of leaf rolling of *SNP*-3A-2 was significantly higher than that of *SNP*-3A-1.

MYB proteins in the plant constitute one of the largest TF families involved in diverse processes, namely, plant growth, development, metabolic and stress responses. Previous studies reported that MYB proteins are involved in leaf rolling regulation. Ectopic expression of *GhMYB7* in *Arabidopsis thaliana* led to leaf rolling by activating a suite of genes involved in secondary cell wall formation, resulting in increased cellulose and lignin content (Huang et al., 2016). A *MYB* gene *NbPHAN* controlled leaf development by suppressing the *NTH20* expression gene and regulating plant tolerance to drought stress. *NbPHAN*-silenced plants exhibited severe downward rolling and an increased rate of water loss (Huang et al., 2013). Here, the leaves of the *tamyb5* mutant did not exhibit a severe curling response to drought stress compared with WT (Figure 2). The results confirmed that *TaMYB5* was involved in leaf rolling regulation of wheat. Moreover, we found that *NRL1* was a downstream target gene of TaMYB5 (Figure 8). *NRL1* plays a critical role in leaf rolling by regulating bubble cell development (Hu et al., 2010). Mature leaves usually showed adaxially leaf rolling under water stress, which may be related to the bulliform cells (Fujino et al., 2008; Li et al., 2010). Under drought stress, dehydration reduces the turgor pressure of bulliform cells, resulting in leaf fold up on both sides and eventually leaf rolling. Once the water is restored, these cells absorb the water and expand again, and the leaves unfold again (Hsiao et al., 1984; Price et al., 1997). Therefore, it is speculated that the regulation of bulliform cells may be an intermediate step of *TaMYB5* in regulating wheat leaf rolling under drought stress.

Leaf rolling limited transpiration and increased canopy temperature under drought stress (Lian et al., 2004; Yan et al., 2009). Canopy temperature is negatively associated with yield under water stress (Melandri et al., 2020). These two traits are important considerations for plant development and drought tolerance. However, few studies addressed functional markers for these traits. Based on the SNP locus of 544 bp in the *TaMYB5* coding region, we developed a functional molecular marker related to leaf rolling and canopy temperature (Figures 3, 4), which could be used for marker-assisted selection for leaf morphology and drought resistance. These results indicate that *TaMYB5* may have multiple effects on plant development and drought tolerance. In addition, we identified two alleles of *TaMYB5*, *SNP*-3A-1, and *SNP*-3A-2, based on the SNP locus of 544 bp. Based on the genotyping analysis of landraces and modern varieties in China, we found that the *SNP*-3A-1 genotype with low CT was positively selected in arid areas, while the *SNP*-3A-2 genotype with high leaf rolling was positively selected in humid areas (Figure 5). This may be due to the different breeding objectives of each wheat zone. The *SNP*-3A-1 genotype in arid areas is associated with increased drought tolerance, while the *SNP*-3A-2 genotype in humid areas is associated with enhanced photosynthesis. It is reported that the genotypes with a high degree of leaf rolling generally have less water uptake in the field under drought stress (Cal et al., 2019). The *SNP*-3A-2 genotype with a high degree of leaf rolling may not be selected in arid areas because it absorbs less water.

We tried to find out the reasons for the differences between the two *TaMYB5* genotypes by yeast one-hybrid and LUC assays, but no difference was found between them. Meta-analysis of large-scale phosphoproteomic data showed protein phosphorylation mainly through forming an ester bond with Ser (80–85%), Thr (10–15%), or Tyr (0–5%) residues and then modifying the proteins in plants (Champion et al., 2004; Wijk et al., 2014). We observed a difference of Thr and Lys in the amino acids of the two proteins of TaMYB5 and then predicted their phosphorylation. Surprisingly, the differences in phosphorylation sites between the two proteins were identified (Supplementary Figure 6). Then, we selected eight accessions for each of the two extreme leaf rolling phenotypes and identified 13 variations in their promoter sequences. We then detected the expression level, but there was no significant difference in their expression levels (Supplementary Figure 7). So we inferred that the difference between the two haplotypes was caused by variations in the coding region rather than that in the promoter. Therefore, we speculated that a certain kinase might affect the phosphorylation of TaMYB5, resulting in different functions of the two proteins, which requires further experimental verification.

**FIGURE 9:**
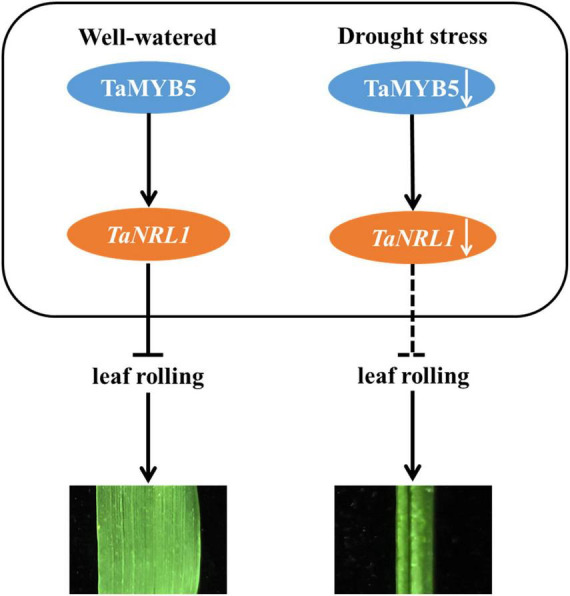
A proposed model for the *TaMYB5* gene in the regulation of adaxial rolling of leaf. Under well-watered, *TaNRL1* was expressed normally and inhibited leaf rolling and leaves showed a flat state. Under drought stress, the expression of *TaNRL1* decreased along with the decrease of *TaMYB5*, which reduced its ability to inhibit leaf rolling. The promoting role is represented by →, and ⊣ shows the inhibiting role.

Overall, our research reveals a possible regulatory network, as shown in Figure 9: *TaMYB5* modulates leaf rolling by promoting the expression of the *TaNRL1* gene in wheat. Under well-watered condition, TaMYB5 did not affect the expression of *TaNRL1*, which inhibited leaf rolling and showed flat leaf. Under drought stress, the expression of *TaMYB5* was reduced, and the expression of *TaNRL1* decreased along with the decrease of *TaMYB5*, which weakened the inhibitory effect of *NRL1* on rolling leaves (Hu et al., 2010), thus leaves showed a rolling state.

Collectively, we identified a novel MYB gene *TaMYB5*, which is a transcriptional activator and regulates leaf rolling by promoting the expression of *TaNRL1*. In addition, *TaMYB5* is also associated with canopy temperature. The functional marker *TaMYB5-3A*-dCAPS will be helpful for future wheat breeding.

## Data availability statement

The original contributions presented in this study are included in the article/Supplementary material, further inquiries can be directed to the corresponding author/s.

## Author contributions

RJ, JgW, CL, and JC conceived the idea. ZZ, JgW, CL, XM, GH, and JpW performed the experiments. ZZ, JgW, and LL analyzed the data. ZZ and JgW wrote the manuscript. RJ, JC, and JgW revised the manuscript. All authors contributed to the article and approved the submitted version.
