# Supplementary material for: A transcription factor TaMYB5 modulates leaf rolling in wheat
Source: Front Plant Sci. 2022 Aug 23;13:897623. doi: 10.3389/fpls.2022.897623 (PMC9445664; doi:10.3389/fpls.2022.897623)
Supplement: Supplementary file 1 [file Data_Sheet_1.docx]

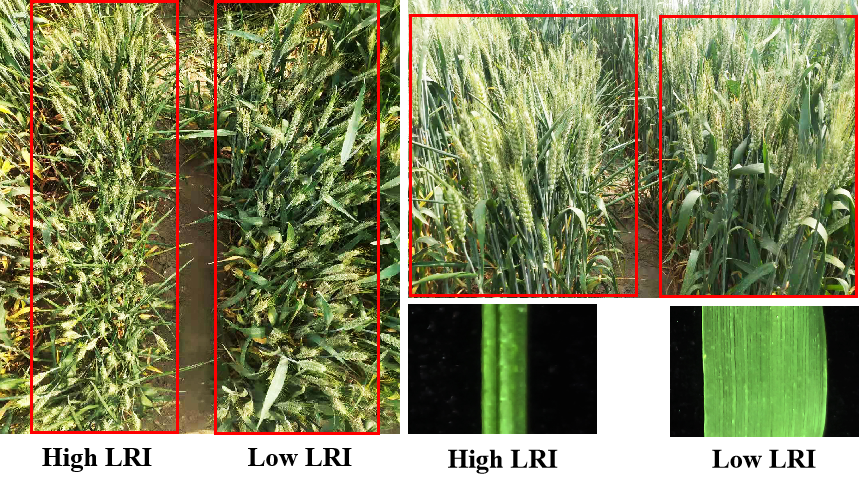


**Supplementary Figure 1** | Two extreme phenotypes of leaf rolling in the Population 1. LRI, leaf rolling index.


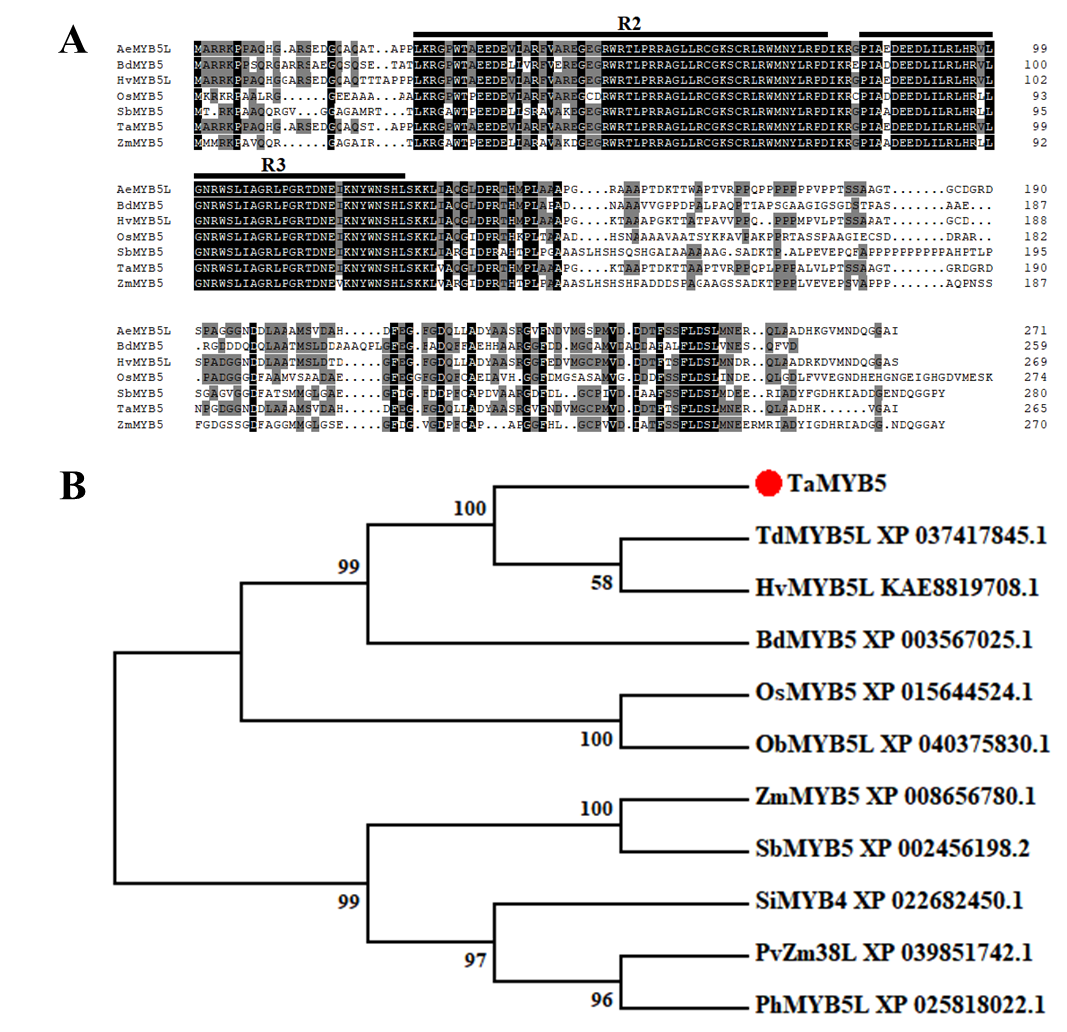


**Supplementary Figure 2** | Structural analysis and phylogenic tree of TaMYB5 protein. **(A)** Amino acid sequences alignment of TaMYB5 and homologous proteins. Amino acids shaded by color are conserved, black shaded amino acids indicate the highest similarity, and gray less. **(B)** Neighbor-joining phylogenic tree of TaMYB5. TaMYB5 was marked with red dots. Ta, *Triticum. aestivum*; Td, *Triticum dicoccoides*; Hv: *Hordeum vulgare*; Bd, *Brachypodium distachyon*; Sb, *Sorghum bicolor*; Zm, *Zea mays*; Si; *Setaria italica*; Ph, *Panicum hallii*; Pv, *Panicum virgatum*; Ob: *Oryza brachyantha*; Os, *Oryza sativa*.


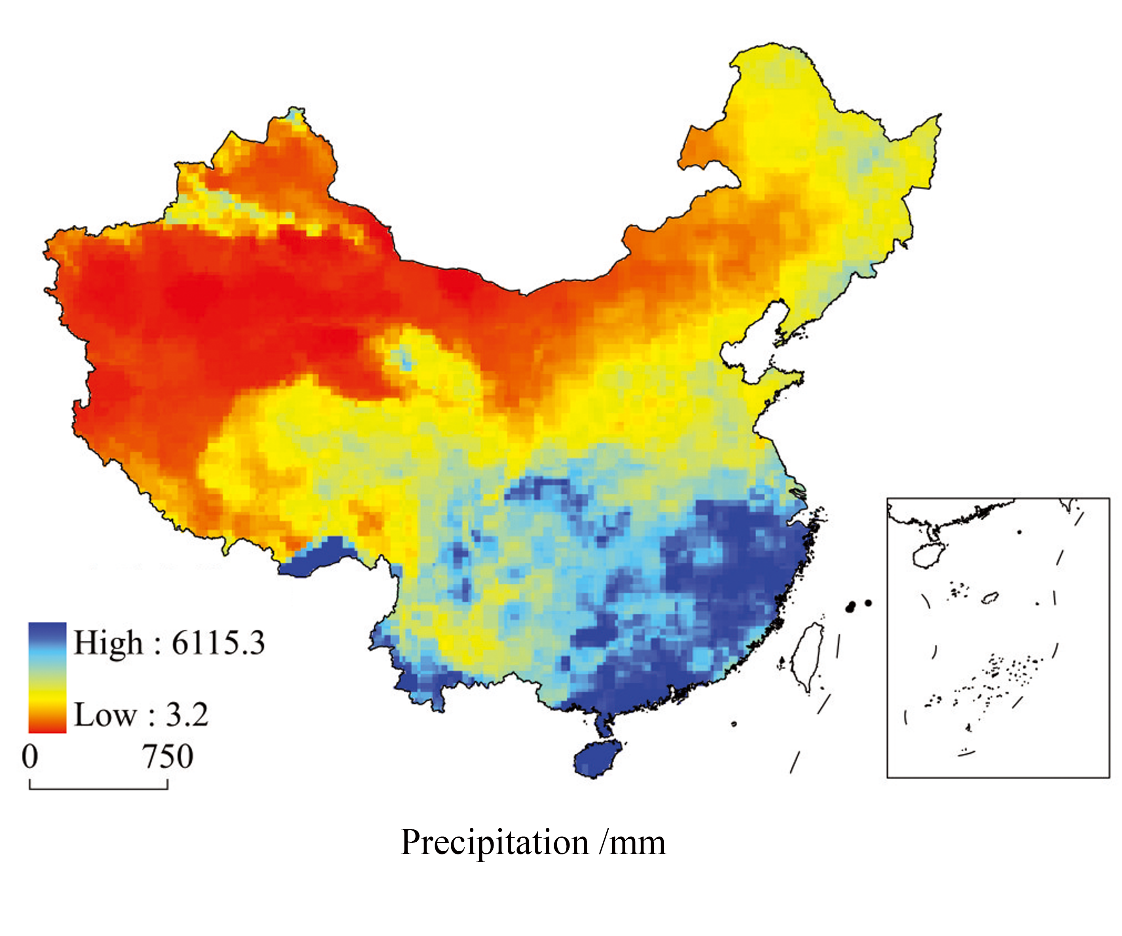


**Supplementary Figure 3** | Annual precipitation map of China. Map from Zeng et al ([2011](#_ENREF_48)).


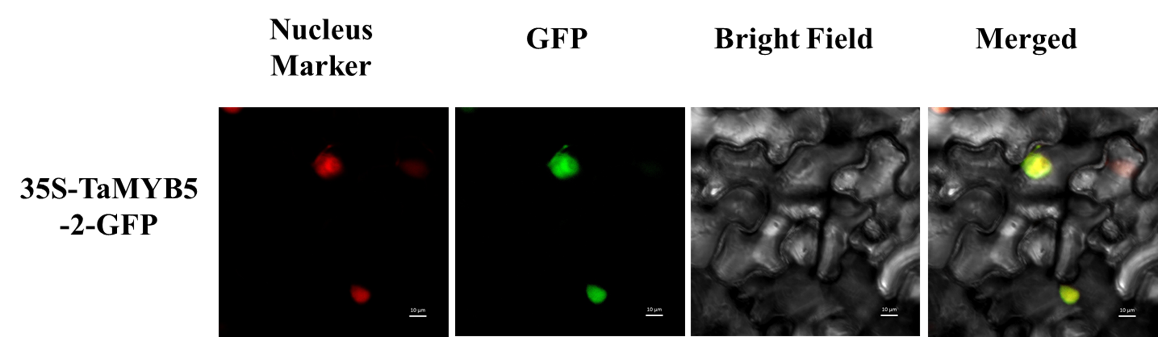


**Supplementary Figure 4** | Subcellular localization of TaMYB5-2. TaMYB5-2 correspond to *SNP*-3A-2 amino acid sequences.

**
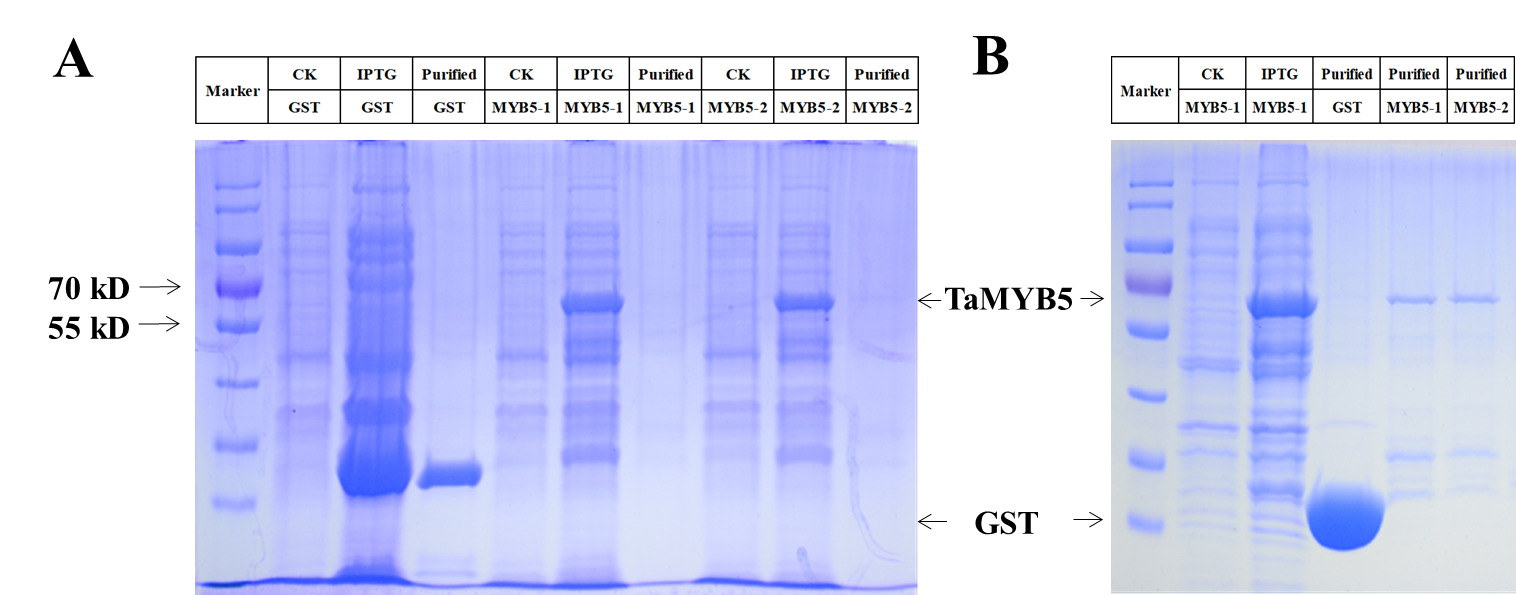
**

**Supplementary Figure 5** | Purification of TaMYB5-GST (MYB5-1 and MYB5-2) and GST proteins. **(A)** Image of protein purification. **(B)** Supplement image of protein. TaMYB5-GST protein was degraded in figure A, so figure B is added as the supplement of figure A. Purified proteins are marked by arrows. TaMYB5-GST and GST proteins were induced by 0.2 mM IPTG at 16°C for 10 h, and it was purified using glutathione-sepharose 4B.


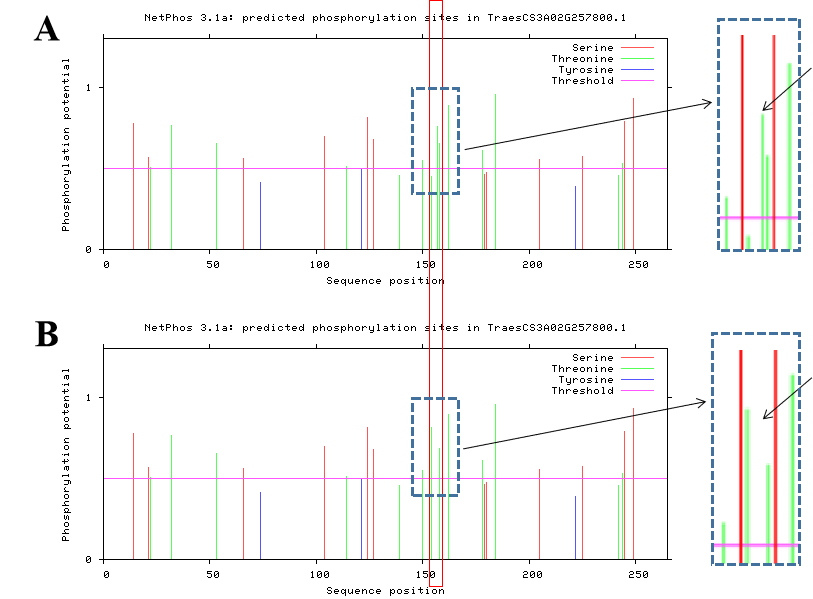


**Supplementary Figure 6** | Prediction of phosphorylation sites for TaMYB5 of both *SNP*-3A-1 **(A)** and *SNP*-3A-2 **(B)**. A partial enlargements of figure A and B are shown at the right. Two arrows on the far right indicate the 157th amino acid site that causes changes in phosphorylation in both proteins. (https://services.healthtech.dtu.dk/service.php?NetPhos-3.1).


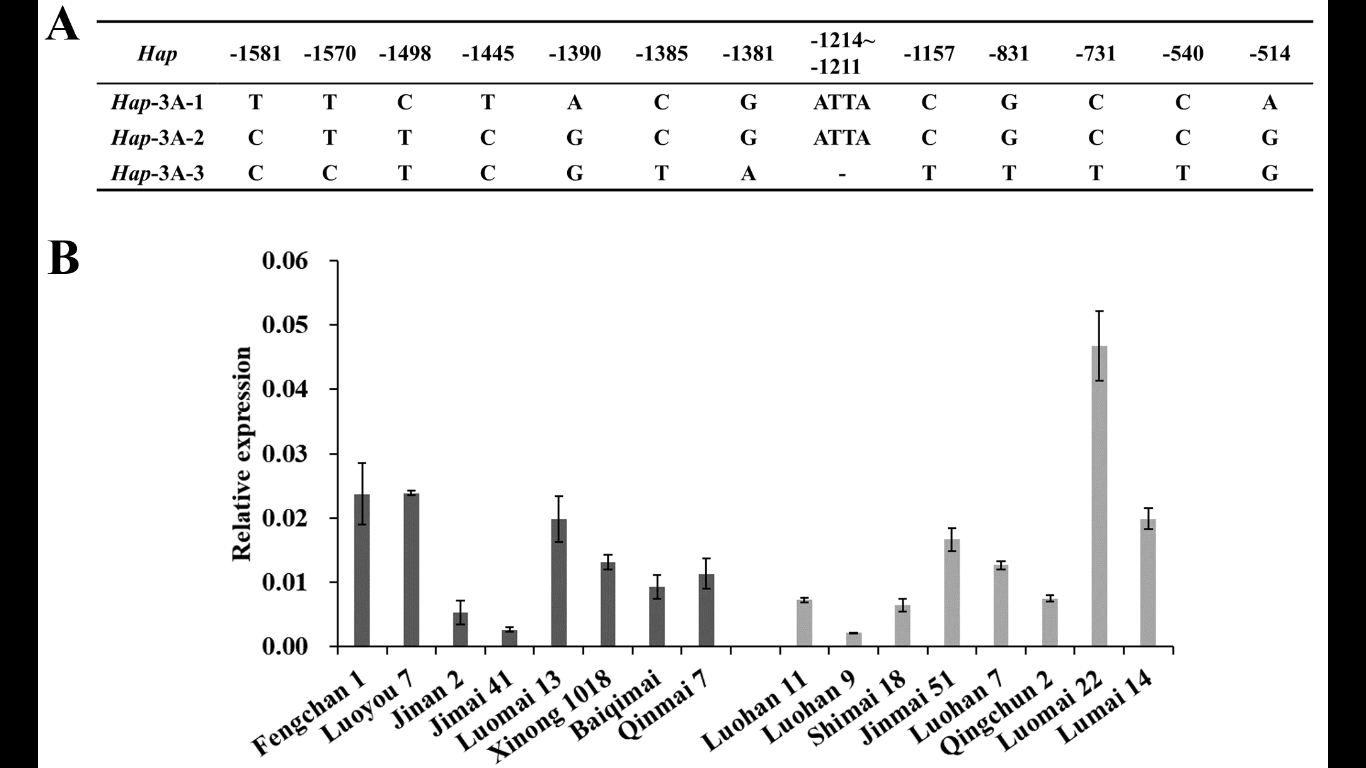


**Supplementary Figure 7** | Promoter variation and expression analysis of *TaMYB5*. **(A)** 13 variations detected in the *TaMYB5* promoter region. **(B)** Expression levels of TaMYB5 in two extreme leaf-rolling accessions. Dark gray indicates the expression level of flat-leaf accession, and light gray presents that of rolling-leaf accession. All data are the means ±SE of three independent experiments.

**Supplementary Table 1 |** Primers used for this study

| **Primer** | **Primer sequence (5′-3′)** | **Experimental purpose** |
| --- | --- | --- |
| TaMYB5-3A-F1 | TGACACGAAATTCCTCCGTTTTC | Specific primers for genome coding regions |
| TaMYB5-3A-R1 | ATTTCGCTAGTCAGCTGATTCGCG | Specific primers for genome coding regions |
| TaMYB5-3A-F2 | GTACTGCTACGTGTCGCTGGT | Specific primers for genome promoter region |
| TaMYB5-3A-R2 | GTCTGTTCCGCTTCCGGAAAACG | Specific primers for genome promoter region |
| TaMYB5-3A-dCAPS-F | ACGGCAGCTCCTACCGACAAGT | Marker development for SNP 544 (C/A) |
| TaMYB5-3A-dCAPS-R | GAGGTCGTCATTGCCTCCGT | Marker development for SNP 544 (C/A) |
| TaNRL1-5A-F | GCCTGTAGCCAGTTGTAGGGGA | Genome-specific primers |
| TaNRL1-5A-R | GCACAACGTCCGGTGATGGAT | Genome-specific primers |
| M13F | TGTAAAACGACGGCCAGT | Sequencing primersfor TaMYB5-3A |
| M13R | CAGGAAACAGCTATGACC | Sequencing primers for TaMYB5-3A |
| TaMYB5-RT-F | AGTCTTCAACGACGTGATGGGGTGT | Real-time PCR |
| TaMYB5-RT-R | GATAGCGCCAACCTTGTGATCAGC | Real-time PCR |
| TaTUBLIN-RT-F | CGTGCTGTCTTTGTAGATCTCG | Real-time PCR |
| TaTUBLIN-RT-R | GACCAGTGCAGTTGTCTGAAAG | Real-time PCR |
| TaMYB5-cDNA-F | AGCCAAAATCCAAAAGCCTCCGC | Genome-specific primers |
| TaMYB5-cDNA-R | GATAGCGCCAACCTTGTGATCAGC | Genome-specific primers |
| TaMYB5-GAL4DB-F | TCTCTAGAACTAGTGGATCCAGCCAAAATCCAAAAGCCTCCGC | Constructing transcriptional activity vector |
| TaMYB5-GAL4DB-R | ATAAGCTTGATATCGAATTCGATAGCGCCAACCTTGTGATCAGC | Constructing transcriptional activity vector |
| TaMYB5-PB42AD-F | TGCCTCTCCCGAATTCAGCCAAAATCCAAAAGCCTCCGC | Constructing yeast one-hybrid vector |
| TaMYB5-PB42AD-R | CGAGTCGGCCGAATTCGATAGCGCCAACCTTGTGATCAGC | Constructing yeast one-hybrid vector |
| TaNRL1-Lacz-F | ATCTGTCGACCTCGAGGCCTGTAGCCAGTTGTAGGGGA | Constructing yeast one-hybrid vector |
| TaNRL1-Lacz-R | GAGCACATGCCTCGAGGCACAACGTCCGGTGATGGAT | Constructing yeast one-hybrid vector |
| TaMYB5-pGEX-F | TGGATCCCCGGAATTCAGCCAAAATCCAAAAGCCTCCGC | Constructing GST-TaMYB5 protein vector |
| TaMYB5-pGEX-R | GGCCGCTCGAGTCGACGATAGCGCCAACCTTGTGATCAGC | Constructing GST-TaMYB5 protein vector |
| Probe-3xAC-F | AGTCCACCTAACGCCACCTAACGCCACCTAACGCTGTT | EMSA probe |
| Probe-3xAC-R | AACAGCGTTAGGTGGCGTTAGGTGGCGTTAGGTGGACT | EMSA probe |
| mProbe-3xAC-F | AGTCCAAATATTGCCAAATATTGCCAAATATTGCTGTT | EMSA mutated probe |
| mProbe-3xAC-R | AACAGCAATATTTGGCAATATTTGGCAATATTTGGACT | EMSA mutated probe |
| TaMYB5-1300-GFP-F | CCAAATCGACTCTAGAAGCCAAAATCCAAAAGCCTCCGC | Constructing subcellular localization vector |
| TaMYB5-1300-GFP-R | TGCTCACCATGGTACCGATAGCGCCAACCTTGTGATCAGC | Constructing subcellular localization vector |
| TaNRL1-0800-F | CGGTATCGATAAGCTTGCCTGTAGCCAGTTGTAGGGGA | Constructing LUC reporter vector |
| TaNRL1-0800-R | TTGGCGTCTTCCATGGGCACAACGTCCGGTGATGGAT | Constructing LUC reporter vector |

**Supplemental Table** **2 |** Annotation of candidate genes in the block of chromosome 3A.

| Candidate gene | Annotation |
| --- | --- |
| *TraesCS3A02G257600* | Type II inositol 3,4-bisphosphate 4-phosphatase |
| *TraesCS3A02G257700* | Potassium channel |
| *TraesCS3A02G257800* | Myb transcription factor |
| *TraesCS3A02G257900* | ENTH/VHS family protein |
| *TraesCS3A02G258000* | ABC transporter B family protein |
| *TraesCS3A02G258100* | GDSL esterase/lipase |
| *TraesCS3A02G258200* | ABC transporter B family protein |
| *TraesCS3A02G258300* | ABC transporter B family-like protein |
| *TraesCS3A02G258400* | ABC transporter B family protein |
| *TraesCS3A02G258500* | 1-aminocyclopropane-1-carboxylate deaminase/D-cysteine desulfhydrase |
| *TraesCS3A02G258600* | Farnesyl diphosphate synthase |
| *TraesCS3A02G258700* | Core-2/I-branching beta-1,6-N-acetylglucosaminyltransferase family protein |

**Supplementary Table 3 |** Details of the AK58 mutants (*tamyb5*).

| **Name** | **Line** | **Chr.** | **Chr position (bp)** | **WT** | **Alt** | **Consequence** | **CDS position (bp)** | **Amino acids** | **Codons** |
| --- | --- | --- | --- | --- | --- | --- | --- | --- | --- |
| M1 | M186 | 3A | 479755545 | C | T | Missense variant | 56 | R/W | CGG/TGG |
| M2 | M451 | 3A | 479755234 | C | T | Missense variant | 135 | L/F | CTC/TTC |
| M3 | M483 | 3A | 479755206 | C | T | Missense variant | 144 | A/V | GCC/GTC |

**Supplementary Table 4 |** The information of thirty-two wheat accessions

| **Number** | **Accession** | **Origin** | **Number** | **Accession** | **Origin** |
| --- | --- | --- | --- | --- | --- |
| 1 | PANDAS | Italy | 17 | Linkang 5108 | Shanxi, China |
| 2 | An85 Zhong124-1 | Beijing, China | 18 | Baiqimai | Gansu, China |
| 3 | Yanzhan 1 | Henan, China | 19 | Changle 5 | Shandong, China |
| 4 | Bawangbian | Hebei, China | 20 | Hongheshang | Shanxi, China |
| 5 | Beijing 10 | Beijing, China | 21 | Beijing 8686 | Beijing, China |
| 6 | Beijing 14 | Beijing, China | 22 | 04-044 | Beijing, China |
| 7 | Cangzhouxiaomai | Hebei, China | 23 | 04-030 | Beijing, China |
| 8 | Changwu 131 | Shaanxi, China | 24 | 9th-25 | CIMMYT |
| 9 | Chang 6878 | Shanxi, China | 25 | Ziganbaimangxian | Henan, China |
| 10 | Dali 1 | Shaanxi, China | 26 | Jingpin 10 | Beijing, China |
| 11 | Dan R8093 | Beijing, China | 27 | 9th-5-1 | CIMMYT |
| 12 | Fengkang 13 | Beijing, China | 28 | 9th-50-1 | CIMMYT |
| 13 | Jimai 41 | Hebei, China | 29 | Neixiang 188 | Henan, China |
| 14 | Jimai 6 | Hebei, China | 30 | Jing 411 | Beijing, China |
| 15 | Jin 2148-7 | Fujian, China | 31 | Chinese Spring | Sichuan, China |
| 16 | Jinghe 8922 | Beijing, China | 32 | Baicaomai | Henan, China |
